# The Book World of Medicine and Science

**Published:** 1905-01-14

**Authors:** 


### The Book World of Medicine and Science.

The Simple Medical Year Book, or Private Medical Ledger. (London: John Bale, Sons, and Danielsson. Price 10s. 6d. net.)

The busy practitioner will find this ledger to be of great use. By keeping it filled in up-to-date he can readily appreciate the exact financial position of his practice at any moment; a desideratum which may at any time through sickness or other causes suddenly develop into an urgent necessity. Incidentally it may be mentioned that the book reduces the laborious calculations ordinarily required in filling up the Income-Tax Assessment Form into the simple task of a few minutes. The ledger consists of separate pages for each week, and each page contains separate columns for fees earned, cash received, surgery expenses, various other professional expenses, and total expenses. At the end are pages for the quarterly totals and the summary for the year. These are followed by indexed blank pages for memoranda. The handy size and the admirable arrangement of the Private Medical Ledger combine to render it essential to the busy general practitioner.

The Schott Treatment for Chronic Heart Diseases. By Richard Greene, F.R.C.P. Edin. Third edition. (London: Scientific Press, Limited. 1904. Pp. 16. Price 6d.)

The Nauheim method of managing patients manifesting evidences of cardiac muscle failure has been proved by experience to be effectual in affording marked alleviation when applied in suitable cases. Although it is many years since the Brothers Schott elaborated their hydrotherapeutic procedures and physical exercises into a definite system, the rationale of the treatment is still ill understood in this country by many practitioners, and the exaggerated claims of some few enthusiasts have tended to quicken the not unnatural prejudice of many conservative physicians to measures which, unless carefully safeguarded, are liable to fall into the hands of mere quacks. Dr. Greene, in his modest pamphlet, writes of the advantages of the so-called Schott treatment from personal experience, and although he does not attempt to explain the physiological and therapeutical bases of the method, yet he succeeds in presenting an attractive and suggestive description of the system, which is sure to be of service to medical practitioners.

The Treatment of Syphilis By F. J. Lambkin, Lieut.-colonel R.A.M.C., specialist at the Army Headquarters, India. (London: Baillière, Tindall and Cox. 1905. Pp. 80. Price 2s. net.)

Though the treatment of syphilis means much more than the administration of mercury, it is natural that the methods of exhibiting this drug and its preparations should occupy a large part of this manual. Rejecting mercurial treatment by the mouth wherever inunction or intramuscular injection is possible the author expresses a marked preference for the last method of which his large experience in India permits him to speak with authority. While it is certain that a large proportion of syphilitics are prone to neglect regular treatment so long as not reminded of their disease by definite symptoms, it is questionable to what extent this depends on the employment of the internal method of administration or how far it would be improved, in civil practice, by a weekly injection at the hands of the medical attendant. In France the intramuscular method is employed as a routine measure to a much greater extent than in this country where it does not appear to have met with a favourable reception. The modification of Lang's mercurial cream recommended by the author seems to offer definite advantages over the soluble salts of mercury, as its injection is less painful and requires less frequent repetition. Some useful hints are given as to the use of iodide of potassium, although the author appears to somewhat under-estimate its value.

Diseases of the Ear. By James Kerr Love, M.D. (Bristol: John Wright and Co. 1904. Pp. 339, with 54 stereograms.)

The number of books on diseases of the ear which have recently made their first appearance is considerable, but it must be confessed that most of them are exceedingly dry and uninteresting to read, and it is therefore with enhanced pleasure that one finds in Dr. Love's book instruction most refreshingly imparted. This quality of freshness is no doubt largely due to the fact that the book is a record of personal experience rather than a bibliographical dictionary like so many of its fellows, but this does not mean that the information it contains is unduly restricted; quite otherwise. The most valuable portions of the book are, we think, the chapter on deaf-mutism and the stereoscopic photographs illustrating the anatomy of the ear. The former includes part of a research conducted by Dr. Love, under the auspices of the Carnegie Trust, for the Universities of Scotland; and in it there is much valuable information concerning the education of deaf-mutes, special attention being drawn to the residual hearing which many of these patients possess. With regard to the stereograms we do not think any praise too great. The book is one which should be studied by every practitioner who is at all interested in ear work.

Manual of Diseases of Women. By H. Macnaughton-Jones, M.D., M.Ch. Ninth Edition. (London: Baillière, Tindall and Cox. 1904. Pp. 1,044. Price 21s. net.)

But little need be said of this edition, except that every care has been evidently taken to bring it quite up to date. A valuable feature is the prolific manner in which the text is illustrated, and it might almost be said that this alone makes the book worth having. The chapters on diseases of the rectum, kidneys, and ureters rather unnecessarily add to the volume of the book, and could have been omitted with considerable advantage.

An Introduction to Dermatology. By Norman Walker, M.D. Third Edition. (Bristol: John Wright and Co. 1904 Price 2s. 6d. net)

This is an excellent text-book, and a very pleasant one to read. It is a simple and clear exposition of dermatology, and is altogether free from redundancies. The only criticism we have to make is that some of the coloured plates, if not definitely misleading, fail to convey accurate representations to the reader. Plate XX., for example, is unlike any case of psoriasis that we have seen, and we should suggest the omission of this plate and one or two of the others from the next edition.

### BOOKS RECEIVED.

Kegan Paul, Trench, Trübner, and Co.
"Fever Nursing." By R. W. Wilcox, M.A.

Swan Sonnenschein and Co., Ltd.
"Premature Burial." By Tebb and Vollum. Second edition.

Edward Arnold.
"Human Embryology and Morphology." By A. Keith, M.D., F.R.C.S.

A. and C. Black.
"Cairo of To-day."

Baillière, Tindall and Cox.

